# Epidemiology of Norovirus Outbreaks Reported to the Public Health Emergency Event Surveillance System, China, 2014–2017

**DOI:** 10.3390/v11040342

**Published:** 2019-04-11

**Authors:** Yiyao Lian, Shuyu Wu, Li Luo, Bin Lv, Qiaohong Liao, Zhongjie Li, Jeanette J. Rainey, Aron J. Hall, Lu Ran

**Affiliations:** 1 Division of Infectious Disease, Key Laboratory of Surveillance and Early-warning on Infectious Disease, Chinese Centre for Disease Control and Prevention, Beijing 102206, China; lian_yiyao@163.com (Y.L.); rosemary214@163.com (L.L.); liaoqiaohong1983@163.com (Q.L.); lizj@chinacdc.cn (Z.L.); 2 Division of Global Health Protection, Center for Global Health, U.S. Centers for Disease Control and Prevention, Beijing 100600, China; kei3@cdc.gov (S.W.); jkr7@cdc.gov (J.J.R.); 3 Xiaogan Center for Disease Control and Prevention, Xiaogan 432000, China; xgmj@163.com; 4 Division of Viral Diseases, National Center for Immunization and Respiratory Diseases, U.S. Centers for Disease Control and Prevention, Atlanta, GA 30333, USA; ajhall@cdc.gov

**Keywords:** norovirus, gastroenteritis, disease outbreaks, China

## Abstract

We conducted a retrospective analysis of norovirus outbreaks reported to the National Public Health Emergency Event Surveillance System (PHEESS) in China from January 1, 2014 to December 31, 2017. We reviewed all acute gastroenteritis outbreaks (*n* = 692) submitted to PHEESS to identify the frequency, seasonality, geographic distribution, setting, and transmission mode of outbreaks due to norovirus. A total of 616 norovirus outbreaks resulting in 30,848 cases were reported. Among these outbreaks, 571 (93%) occurred in school settings including 239 (39%) in primary schools, 136 (22%) in childcare facilities, and 121 (20%) in secondary schools. The majority of outbreaks (63%) were due to person-to-person transmission, followed by multiple modes of transmission (11%), foodborne (5%) and waterborne (3%) transmission. These findings highlight the importance of improving hand hygiene and environmental disinfection in high-risk settings. Developing a standard and quantitative outbreak reporting structure could improve the usefulness of PHEESS for monitoring norovirus outbreaks.

## 1. Introduction

Noroviruses are a leading cause of acute diarrheal illness globally and are associated with almost 50% of all-cause acute gastroenteritis (AGE) outbreaks [1,2]. The World Health Organization (WHO) estimated that noroviruses cause 685 million cases of diarrhea and 212,489 deaths annually [3]. Noroviruses are primarily transmitted via the fecal–oral route including direct person-to-person transmission or indirectly through contaminated food, water, or environmental surfaces [4]. Due to the low infectious dose (18–2800 viral particles needed to infect a healthy adult) and prolonged duration (4 weeks on average; range of 13–56 days) of viral shedding, noroviruses are highly contagious and can result in large outbreaks [2,5,6,7,8]. Norovirus outbreaks occur frequently in semi-closed settings such as restaurants, hospitals, nursing homes for the elderly, schools and day care centers, military-camps, and cruise ships. Although norovirus has been well described as a cause of epidemic AGE in all age groups in many countries [1,4,9], the majority of morbidity and mortality occurs among children and older adults. 

In China, estimates suggest that the incidence rate of norovirus is six cases per 100 person-years in the general population and 16 cases per 100 person-years in children less than 5 years of age [10]. To monitor and respond to big and/or severe outbreaks including those resulting from norovirus, the National Health Commission in China developed the web-based Public Health Emergency Event Surveillance System (PHEESS) in 2004. Clinical hospitals and local public health authorities (each known as a local Center for Disease Control and Prevention [CDC]) at the city, district, and county levels are required to report public health emergency events such as infectious disease outbreaks (i.e., a large number of cases from any disease with known or unknown causes), severe poisonings, and other events that are likely to result in harm or danger to the public to PHEESS [11]. According to the National Guidelines for Information Reporting Management of Public Health Emergency Events, AGE outbreaks involving 20 or more cases, or involving one or more outbreak-related deaths, should be reported to PHEESS [12]. Information on strain subtyping or genotyping is generally not included in the system. Although several studies have described the strain distribution of noroviruses in China [9,13,14,15], little information is available on the epidemiologic characteristics of norovirus outbreaks that occur in China. Monitoring norovirus outbreaks can help to improve our understanding of norovirus epidemiology, identify changes in occurrence, and provide evidence for targeted intervention strategies. 

## 2. Materials and Methods

### 2.1. Data Collection

We conducted a retrospective analysis of norovirus outbreaks reported to PHEESS in China from January 1, 2014 to December 31, 2017. Outbreak reports include information on setting, number of cases (and exposed persons), transmission mode, and onset dates of the first and last detected cases submitted by local CDCs to the structured PHEESS web-based database. Additional information on the sources and procedures used for outbreak detection and investigation, types and results of laboratory tests performed, and outbreak outcomes and control measures is provided in an unstructured narrative with each PHEESS report. The format and structure, completeness, and quality of these narratives vary greatly depending on the local CDC. For this analysis, we downloaded information from all AGE outbreaks that were reported to the structured database from January 1, 2014 to December 31, 2017. We reviewed each narrative report to abstract the etiology of each AGE outbreak and to verify other outbreak information. When inconsistencies were noted between the structured database and data abstracted from the narratives, we used information from the narratives for this analysis. Data for each outbreak (downloaded and abstracted data) were entered into an Excel spreadsheet using a unique outbreak number. We described the usefulness of PHEES for monitoring norovirus outbreaks according to the public health resources that were required to conduct this analysis as well as the completeness of key reporting variables.

### 2.2. Outbreak Definitions

An AGE case is defined as a person with diarrhea (three or more loose stools) or vomiting two or more times in a 24-hour period [16]. AGE outbreaks reported to PHEESS include outbreaks involving 20 or more cases of AGE (with the exception of cholera, dysentery, typhoid, and paratyphoid fever, as they follow a more strict reporting criteria), or one or more outbreak-related deaths occurring in a defined setting (e.g., school, kindergarten, community, or building site). AGE outbreaks including two or more cases of laboratory confirmed (by ELISA or RT-PCR) norovirus infections are defined as norovirus outbreaks [17]. For our analysis, we defined the outbreak onset date and time as the date and time of the first case associated with the outbreak, and outbreak duration as the number of days between the onset date of the first case and the last identified case. The attack rate for each outbreak was defined as the number of cases associated with the outbreak divided by the total number of exposed persons (determined by local staff during the investigation) that was reported to PHEESS. The transmission mode was determined by each reporting site based on the local public health investigation and national guidelines [17] (Appendix A).

### 2.3. Data Management and Analysis

AGE outbreak data were imported into SPSS 17.0 (IBM, Armonk, NY, USA) for data cleaning (i.e., internal consistency) and analysis. We generated descriptive statistics on reported AGE and norovirus outbreaks including the geographic and temporal distribution, setting, and transmission mode of reported outbreaks. The Mann–Whitney U test was used to compare the median attack rates among the norovirus outbreaks caused by a single pathogen and multiple pathogens. We used the Kruskal–Wallis H test to compare the overall median outbreak size and outbreak duration by transmission mode and setting, and the Nemenyi test to compare the median outbreak size and duration between each pair of transmission mode and setting. 

We used ArcGIS 10.3 (Esri, Redlands, CA, USA) to map the annual reporting rates of norovirus outbreaks by province. Population-based reporting rates were calculated for each province using data from the China Census Bureau’s Population Census conducted in 2016 and expressed as outbreaks per 10 million population per year. We calculated the public health resources required to conduct a descriptive analysis of norovirus outbreaks reported to PHEESS based on staff time used for abstracting relevant data from the qualitative narratives. We also described the completeness of the key data elements (i.e., onset date, setting, and transmission mode) from outbreak reports according to the percentage of outbreak reports that included these elements. Statistical significance was assessed using an alpha level of 0.05 for all analyses.

### 2.4. Ethics Statement

This project involved a retrospective analysis of previously collected aggregate outbreak data. China CDC approved this project as a routine surveillance activity. US CDC approved the project as non-research. No personal identifying information was analyzed as part of this project.

## 3. Results

From January 2014 to December 2017, 692 AGE outbreaks were reported to PHEESS. These outbreaks were associated with 33,799 illnesses and three deaths (Table 1). Among the deaths, two patients died of rotavirus infection and one patient died of an unknown etiology. Among all outbreaks, 635 (91.8%) were caused by a single etiology, eight (1.2%) by multiple etiologies, and 49 (7.1%) by unknown etiology. Among the 692 AGE outbreaks, 616 (89.0%) were caused by norovirus, resulting in 30,848 illnesses. No norovirus-associated deaths were reported. The median attack rates of outbreaks caused by a single pathogen tended to be lower than outbreaks caused by multiple pathogens (with 3.7 and 10.4, respectively), but this did not reach statistical significance (Mann–Whitney U = 1630, *p* = 0.08).

Although local CDCs were only required to report outbreaks involving 20 or more cases, 97 (15.7%) norovirus outbreaks with fewer than 20 cases were reported voluntarily. Except for the size and shorter duration, the 97 small outbreaks and the 519 large outbreaks were epidemiologically similar (i.e., seasonal and geographic distribution) (Tables S1 and S2, and Figures S1 and S2). The median size of all reported norovirus outbreaks was 34 cases (interquartile range [IQR]: 23–60 cases) (Table 2). The largest outbreak, involving 753 cases, occurred at a university. Among all norovirus outbreaks, 487 (79%) reported the onset dates for both the first and last cases. The median outbreak duration of these outbreaks was 4.7 days (IQR: 2.3–8.5 days). 

**Figure 1:**
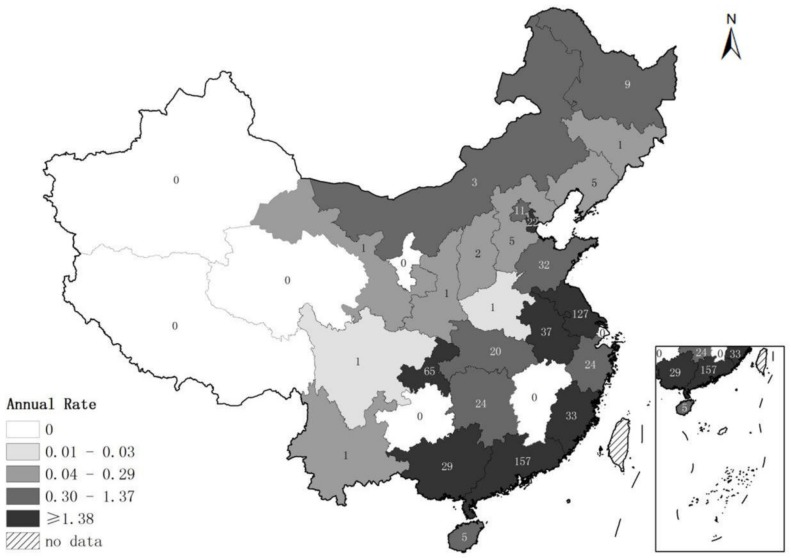
Annual rate of reported norovirus outbreaks per 10 million population by province in China, 2014–2017. The number given in each province indicates the total number of outbreaks over the 4-year study period; the shading denoted by the legend indicates the reporting rate by quartiles.

From 2014 to 2017, 24 (77.4%) of the 31 provinces in mainland China reported at least one norovirus outbreak (Table 2). The number of provinces reporting at least one outbreak increased from ten provinces in 2014 to 14 provinces in 2015 and 2016, and to 21 provinces in 2017. The total number of reported norovirus outbreaks increased from 58 in 2014 to 323 in 2017, and the number of outbreak-associated illnesses increased from 4672 to 15,062 during the same time period. Most norovirus outbreaks were reported by provinces in the eastern and southern regions in China. Among the 616 norovirus outbreaks, 419 (68.0%) were reported by five provinces including Guangdong (*n* = 157), Jiangsu (*n* = 127), Chongqing (*n* = 65), Anhui (*n* = 37), and Fujian (*n* = 33). Norovirus outbreak reporting rates were highest in Chongqing (5.4 outbreaks per 10 million population), followed by Jiangsu (4.0 outbreaks per 10 million population), Guangdong (3.6 outbreaks per 10 million population), and Tianjin (3.6 outbreaks per 10 million population) (Figure 1).

**Figure 2:**
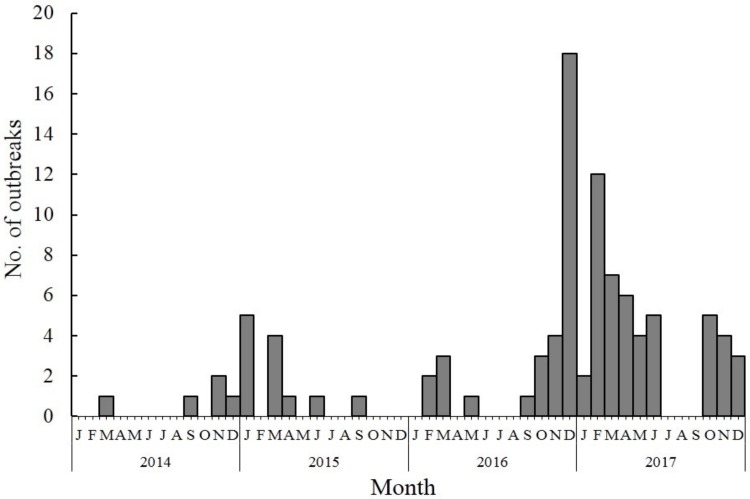
Number of reported norovirus outbreaks by month in China, 2014–2017.

Norovirus outbreaks were reported throughout the year. The majority (*n* = 47, 76.5%) of norovirus outbreaks occurred during October–March, indicating a winter seasonality. Outbreaks were less common during the summer months, particularly in July and August, during which only four (0.7%) of all outbreaks were reported. The number of norovirus outbreaks increased sharply in November 2016, peaking (*n* = 91) in February 2017, which was the highest monthly number of reported outbreaks during the study period (Figure 2).

Among the 616 reported norovirus outbreaks, 387 (62.8%) were caused by person-to-person transmission, 29 (4.7%) were foodborne, and 21 (3.4%) were waterborne (Table 3). Outbreaks most frequently occurred in primary schools (*n* = 239, 38.8%), followed by childcare facilities (*n* = 136, 22.1%), secondary schools (*n* = 121, 19.6%), universities (*n* = 39, 6.3%), and other schools (*n* = 36, 5.8%). The size and duration of norovirus outbreaks varied by setting and transmission mode (all comparisons, *p* < 0.05). Foodborne norovirus outbreaks were significantly larger than those caused by person-to-person transmission (*p* = 0.01). Among the school outbreaks, the size and duration of the outbreak increased with student age (i.e., childcare facility < primary < secondary and university [all comparisons, *p* < 0.05]).

**Figure 3:**
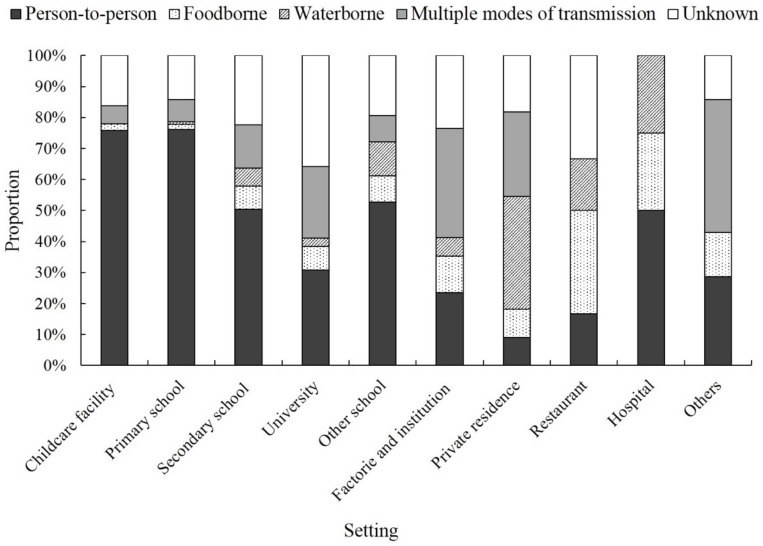
Percentage of norovirus outbreaks by setting attributed to transmission mode, China, 2014–2017. Other schools included vocational schools (12), training organizations (1), and comprehensive schools that consisted of one or more child-care facility, primary school, and secondary school (26). Others included tourism resorts (2), passenger liners (2), prisons (1), nursing homes for the elderly (1), children’s homes (1).

The reported transmission mode varied by outbreak setting (*p* < 0.05). Person-to-person transmission was the primary cause of outbreaks in childcare facilities (75.7%), primary schools (76.1%), and secondary schools (50.4%). Waterborne transmission was more common in private residences, while foodborne transmission was most frequently associated with outbreaks occurring at restaurants (Figure 3). All narratives for norovirus outbreaks included information on control measures. The most common control measures were patient quarantine, environmental disinfection, improving food and water hygiene, and health education.

## 4. Discussion

PHEESS is currently the national system for reporting outbreaks and other public health events in China. Among the 692 reported AGE outbreaks during 2014–2017, 89% were caused by norovirus infections. This finding is consistent with outbreak data from many developed countries. More than 85% of all nonbacterial AGE outbreaks, for example those reported in European countries from 1995 to 2000, were the result of noroviruses [18]. Similarly, noroviruses cause approximately two-thirds all AGE outbreaks with a known etiology in the United States [7]. 

The observed increase of many gastrointestinal diseases including noroviruses during the winter months has been associated with lower temperatures and greater rainfall [19]. Similar to other countries in the Northern Hemisphere, norovirus outbreaks in China generally peaked in winter and early spring (between October and March) [20]. The high reporting rate of norovirus outbreaks in eastern and southern China may partially reflect these environmental factors [20]. Higher rates could also reflect social mixing patterns or the higher population density in the eastern and southern provinces, where higher contact rates and crowding can facilitate person-to-person transmission [19]. This has been proposed as a factor that contributes to norovirus outbreaks in closed populations. In our study, norovirus outbreaks decreased during the winter holidays when schools were closed, reducing contact rates and the risk of transmission in these settings. Additionally, residents in coastal provinces in eastern and southern China (e.g., Guangdong, Jiangsu, Zhejiang) have greater access to seafood including shellfish, which are important reservoirs of norovirus [17,21]. The geographic variability in norovirus outbreak reporting rates in our analysis may also reflect relatively stronger surveillance and reporting practices for AGE outbreaks in the eastern and southern provinces when compared to other regions of the country. We observed an increase in the number of provinces reporting norovirus outbreaks over the 4-year project period, indicating possible improvements in outbreak surveillance and reporting. More work is needed to evaluate the role of reporting practices, climate, and crowding on the observed geographic distribution of norovirus outbreaks in our study.

Most (63.0%) norovirus outbreaks included in our analysis were associated with person-to-person transmission, which is consistent with previous studies in the United States and other countries [4,7]. The low infectious dose and environmental persistence of the norovirus increases secondary transmission through contaminated fomites, hands, and other forms of contact [4]. Narratives from PHEESS documented that vomit from ill students was often handled by classmates during the cleaning process, and this likely contributed to secondary transmission. Prolonged viral shedding including from asymptomatic infections, along with limited long-term immunity, also increases the potential risk of secondary spread through person-to-person transmission [6]. 

Schools (primary, secondary, colleges, and other schools) and childcare facilities accounted for 93% of all the norovirus outbreaks reported to PHEESS. A higher percentage of norovirus outbreaks among school and childcare settings was also reported in Japan, Taiwan, and Hong Kong [22,23,24]. This differs, however, from the United States and Europe where acute and long-term healthcare facilities (e.g., nursing homes) were reported as the most common setting for norovirus outbreaks [25,26,27]. This difference in the primary outbreak setting may reflect the greater number and size of long-term care facilities in the United States and Europe compared to China [25,26,27]. With a mean of 30–50 students per classroom, kindergartens and schools are likely more densely populated in China than in other countries. Close contact between students can facilitate person-to-person transmission, particularly among young children with lower levels of hand hygiene [28]. Some schools in China also do not have regular access to treated drinking water, which leads to potential exposure opportunities to noroviruses [29]. Additionally, the higher reporting rates of school outbreaks may also be an artefact of surveillance and reporting bias. Since 2006, the China Ministry of Health and the Ministry of Education have required school officials to check and screen children attending kindergarten, primary, and middle schools each morning for fever, vomiting, or diarrhea [30]. This program has been helpful in detecting some communicable diseases including norovirus outbreaks occurring at kindergartens and schools. The high incidence of norovirus outbreaks at schools resulting from person-to-person transmission underscores the importance of excluding ill persons, improving hand hygiene, and proper environmental disinfection [2].

Human noroviruses are genetically highly diverse and constantly evolving, resulting in the emergence of new genotypes every 2–4 years [4]. Although PHEESS does not require genotype information, national and some provincial CDCs voluntarily conduct norovirus genotyping (but the data are not reported into PHHESS). According to laboratory analyses of norovirus outbreaks from other published studies, GII.P17-GII.17 was found to be the predominant genotype in 2014–2015, GII.P17-GII.17 and GII.P12-GII.3 in 2015–2016, and GII.P16-GII.2 for 2016–2017 in China [31,32]. From December 2016 to May 2017, the peak of reported norovirus outbreaks was very likely associated with the emerging genotype GII.P16-GII.2 [32]. These observations are consistent with data from other countries, particularly in Asia, where in the winter of 2014, GII.17 became the predominant genotype [33,34,35,36], and in the winter of 2016, GII.P16-GII.2 emerged in China and elsewhere [31,37,38,39,40].

There are several limitations in our analysis. First, many norovirus outbreaks are likely to have not been reported to PHEESS. For example, no norovirus outbreaks were reported from seven provinces during the 4-year study period. This could reflect a low occurrence of outbreaks in these provinces, but could also suggest lower surveillance and reporting capacities, as noted above. Second, although 97 outbreaks involving fewer than 20 cases were reported, PHEESS only requires local CDCs to report large or severe outbreaks. A reporting system that captures data from all norovirus outbreaks (e.g., ≥2 laboratory confirmed cases, regardless of total case count) could help to more fully characterize the epidemiology of norovirus and the effectiveness of control measures in China. Third, epidemiological information collected by PHEESS was limited and not fully standardized; missing and inconsistent data were noted. Abstracting information from the narratives was inefficient, decreasing the overall utility of the system. Additionally, gender and age information are not required for outbreak reports, and both demographic characteristics are useful for identifying risk factors and appropriate control measures. Finally, outbreak reporting guidelines do not include standard instructions for calculating the number of exposed persons. This may have negatively impacted the accuracy of attack rates reported in the system.

## 5. Conclusions

Based on our analysis of PHEESS, norovirus was the most common cause of reported AGE outbreaks in China, resulting in more than 30,000 illnesses during 2014–2017. Outbreak reporting increased during the study period, and the majority of reported outbreaks occurred at childcare facilities and schools, which were most often associated with person-to-person transmission. Our findings highlight the importance of strengthening outbreak surveillance, improving hand hygiene and environmental disinfection, and excluding ill people in these settings. Developing a standard and quantitative outbreak reporting structure could improve the usefulness of PHESS for monitoring norovirus outbreaks.

## Figures and Tables

**Table 1:** Etiology of 692 reported acute gastroenteritis outbreaks in China, 2014–2017.

Etiology	No. of Outbreaks (%)	Total No. of Cases	Total No. of Persons Exposed	Attack Rate (%), Median (IQR)	Total No. of Deaths
**Single etiology outbreaks**	635 (91.8)	31,313	1,331,092	3.7 (1.8, 8.0)	2
Norovirus	611 (88.3)	30,249	1,245,725	3.7 (1.8, 8.0)	0
Non-norovirus ^1^	24 (3.5)	1064	85,367	3.7 (1.7, 8.8)	2
**Multiple etiology outbreaks**	8 (1.1)	993	13,905	10.4 (5.0, 17.6)	0
Norovirus and other etiologies ^2^	5 (0.7)	599	9718	8.6 (6.1, 17.5)	0
Etiologies other than norovirus ^3^	3 (0.4)	394	4187	12.3 (6.9, 15.7)	0
**Not identified**	49 (7.1)	1493	47,608	5.1 (1.9, 10.1)	1
**Total**	692	33,799	1,392,605	3.8 (1.8, 8.3)	3

^1^ Non-norovirus included Salmonella spp., nontyphoidal (9), rotavirus (4), sapovirus (3), enteroadherent E. coli (3), enterotoxigenic E. coli (1), Shigella (1), Shigella flexneri (1), Klebsiella pneumoniae (1), Vibrio parahaemolyticus (1), and Staphylococcus aureus (1). Salmonella spp., nontyphoidal includes all serotypes other than Typhi. ^2^ Norovirus and other etiologies included norovirus and sapovirus (1), norovirus and Shigella (1), norovirus and Salmonella spp., nontyphoidal (1), norovirus and rotavirus (1), and norovirus and enteroadherent E. coli (1). ^3^ Etiologies other than norovirus included astrovirus and Shigella (1), Shigella and enteroinvasive E. coli (1), and rotavirus and sapovirus (1).

**Table 2:** Number of reported norovirus outbreaks and outbreak-associated illnesses in China, 2014–2017.

Year	Total No. of Outbreaks	Total No. of Cases	Total No. of Deaths	No. of Reported Provinces	Outbreak Size (cases), Median (IQR)	Outbreak Duration (days), Median (IQR) ^1^
2014	58	4672	0	10	48 (26, 91)	5.9 (2.8, 8.1)
2015	100	5861	0	14	39 (23, 63)	5.8 (2.6, 8.2)
2016	135	5253	0	14	31 (21, 49)	4.0 (2.0, 8.6)
2017	323	15,062	0	21	34 (24, 58)	4.8 (2.3, 8.8)
Total	616	30,848	0	24	34 (23, 60)	4.7 (2.3, 8.5)

^1^ N = 487 outbreaks.

**Table 3:** Number of reported norovirus outbreaks and outbreak-associated indicators by transmission mode and setting in China, 2014–2017.

Outbreak Characteristic	No. of Total Outbreaks	No. of Total Cases	Attack Rate (%), Median (IQR)	Outbreak Size (cases), Median (IQR) ^1^	Outbreak Duration (days), Median (IQR) ^2^
**Transmission Mode**					
Person-to-person	387 (62.8)	16,884	3.6 (1.9, 7.5)	31 (22, 52)	5.3 (2.6, 8.9)
Foodborne	29 (4.7)	1947	7.8 (2.2, 18.9)	53 (38, 86)	3.4 (1.3, 5.4)
Waterborne	21 (3.4)	1616	3.6 (1.2, 5.9)	47 (31, 107)	6.9 (3.6, 9.2)
Multiple	66 (10.7)	4665	4.9 (1.4, 9.5)	48 (26, 84)	4.5 (2, 8.1)
Unknown	113 (18.3)	5736	3.3 (1.6, 8.3)	38 (24, 67)	3.4 (2, 7.3)
**Exposure Setting**					
Childcare facility	136 (22.1)	3639	8.4 (5.2, 16.2)	24 (17, 32)	2.3 (1.2, 4.7)
Primary school	239 (38.8)	10,353	2.9 (1.7, 5.7)	34 (23, 54)	5 (2.4, 8.2)
Secondary school	121 (19.6)	7586	2.7 (1.5, 5.6)	52 (34, 74)	7.4 (4.2, 12.8)
University	39 (6.3)	5081	1.1 (0.6, 2.1)	79 (57, 139)	10.4 (7.3, 16.3)
Other school ^3^	36 (5.8)	1819	2.8 (1.3, 5.3)	36 (25, 61)	3.3 (2.6, 9.5)
Factory and institute	17 (2.8)	799	7.8 (4.2, 15.9)	34 (29, 71)	2.7 (1.1, 7.6)
Restaurant	6 (1.0)	524	24.2 (7.5, 40.7)	70 (29, 164)	-
Private residence	11 (1.8)	456	3.2 (0.7, 8.0)	37 (27, 42)	6.6 (2.4, 9.6)
Hospital	4 (0.6)	297	14.8 (6.4, 46.4)	55 (27, 142)	-
Others^ 4^	7 (1.1)	294	10.0 (2.2, 18.2)	28 (20, 67)	2.6 (1.8, 4.7)

^1^ Outbreak size was significantly different between different transmission modes and exposure settings by the Kruskal–Wallis H test (*p* < 0.05). ^2^ Outbreak duration was significantly different between the different transmission modes and exposure settings by the Kruskal–Wallis H test (*p* < 0.05), *n* = 487 outbreaks. ^3^ Other schools included vocational schools (12), training organizations (1), comprehensive schools that consisted of one or more child-care facility, primary school, secondary school (26). ^4^ Others included tourism resorts (2), passenger liners (2), prisons (1), nursing homes for the elderly (1), and children’s homes (1).

## Supplementary Materials

The following are available online at https://www.mdpi.com/1999-4915/11/4/342/s1, Figure S1: (**A**) Monthly distribution of norovirus outbreaks involving ≥20 cases reported to the Public Health Emergency Event Surveillance System (PHEESS), China, 2014–2017 (*n* = 519). (**B**) Monthly distribution of norovirus outbreaks involving <20 cases reported to the Public Health Emergency Event Surveillance System (PHEESS), China, 2014–2017 (*n* = 97); Figure S2: (**A**) Number of norovirus outbreaks involving ≥20 cases reported to the Public Health Emergency Event Surveillance System (PHEESS) by province, China, 2014–2017 (*n* = 519). (**B**) Number of norovirus outbreaks involving <20 cases reported to the Public Health Emergency Event Surveillance System (PHEESS) by province, China, 2014–2017 (*n* = 97); Table S1: Size and duration of norovirus outbreaks reported to the Public Health Emergency Event Surveillance System (PHEESS), China, 2014–2017; Table S2: Transmission mode and setting of norovirus outbreaks reported to the Public Health Emergency Event Surveillance System (PHEESS), China, 2014–2017.

## Appendix A

**Definition of transmission modes in norovirus outbreaks reported to the Public Health Emergency Event Surveillance System (PHEES), China.**

Based on national guidelines [17], the transmission mode of norovirus for each outbreak was assigned into one of the following mutually exclusive categories: person-to-person, foodborne, waterborne, or multiple transmission modes.

Person-to-person transmission is defined as transmission through direct contact with an infected person, their bodily fluids, or their contaminated environments.

Foodborne transmission is defined as transmission by consuming contaminated food. Contamination by human excreta or other substances (such as water) containing norovirus may occur during the production, processing, transportation, or distribution of food. Food contamination with norovirus by infected food workers in the preparation and serving of food was also included in foodborne transmission.

Waterborne transmission is defined as transmission by consumption of contaminated of drinking water sources such as bottled water, municipal water supply, well water, etc., or by exposure to contaminated water from swimming pools and other recreational venues.

Multiple transmission mode is defined as two or more of the above transmission modes in a single outbreak.
