# Supplementary material for: Epidemiology of Norovirus Outbreaks Reported to the Public Health Emergency Event Surveillance System, China, 2014–2017
Source: Viruses. 2019 Apr 11;11(4):342. doi: 10.3390/v11040342 (PMC6520956; doi:10.3390/v11040342)
Supplement: Supplementary file 1 [file viruses-11-00342-s001.pdf]

Table S1. Size and duration of norovirus outbreaks reported to the Public Health Emergency Event Surveillance System (PHEESS), China, 2014–2017

Year	No. of Outbreaks		Total No. of Cases		No. of Reporting Provinces		Outbreak Size (cases), Median (IQR) [1]		Outbreak Duration (days), Median (IQR) [2]	
	A [3]	B [4]	A [3]	B [4]	A [3]	B [4]	A [3]	B [4]	A [3]	B [4]
2014	53	5	4,599	73	10	2	53 (29, 94)	13 (12, 18)	5.9 (2.8, 8.2)	-
2015	88	12	5,679	182	12	5	42 (30, 69)	17 (12, 18)	6 (3.5, 8.4)	2.5 (1.5, 5.0)
2016	103	32	4,824	429	14	5	38 (27, 60)	14 (10, 16)	4.9 (2.5, 9.8)	2 (1.2, 4.0)
2017	275	48	14,400	662	19	12	40 (28, 65)	14 (12, 17)	5.5 (2.6, 9.8)	2.6 (1.5, 4.0)
**Total**	519	97	29,502	1,346	22	13	40 (28, 66)	14 (12, 17)	5.7 (2.7, 9.3)	2.6 (1.3, 4.0)

[1] Outbreak size was significantly different between A and B by Mann-Whitney U test ($p < 0.001$).

[2] Outbreak duration was significantly different between A and B by Mann-Whitney U test ($p < 0.001$).

[3] For outbreaks involving ≥20 cases.

[4] For outbreaks involving < 20 cases.

Table S2. Transmission mode and setting of norovirus outbreaks reported to the Public Health Emergency Event Surveillance System (PHEESS), China, 2014–2017

Outbreak Characteristic	No. of Outbreaks (%)		No. of Total Cases	
	A [1]	B [2]	A [1]	B [2]
**Transmission Mode**				
Person-to-person	316 (60.9)	71 (73.2)	15,896	988
Foodborne	28 (5.4)	1 (1)	1,942	5
Waterborne	19 (3.7)	2 (2.1)	1,580	36
Multiple	59 (11.4)	7 (7.2)	4,570	95
Unknown	97 (18.7)	16 (16.5)	5,514	222
**Exposure Setting**				
Childcare facility	92 (17.7)	44 (45.4)	3042	597
Primary school	199 (38.3)	40 (41.2)	9791	562
Secondary school	115 (22.2)	6 (6.2)	7502	84
University	39 (7.5)	-	5081	-
Other school [c]	30 (5.8)	6 (6.2)	1727	92
Factory and institute	17 (3.3)	-	799	-
Restaurant	6 (1.2)	-	524	-
Private residence	11 (2.1)	-	456	-
Hospital	4 (0.8)	-	297	-
Others	6 (1.2)	1 (1)	283	11

[1] For outbreaks involving ≥20 cases.

[2] For outbreaks involving < 20 cases.

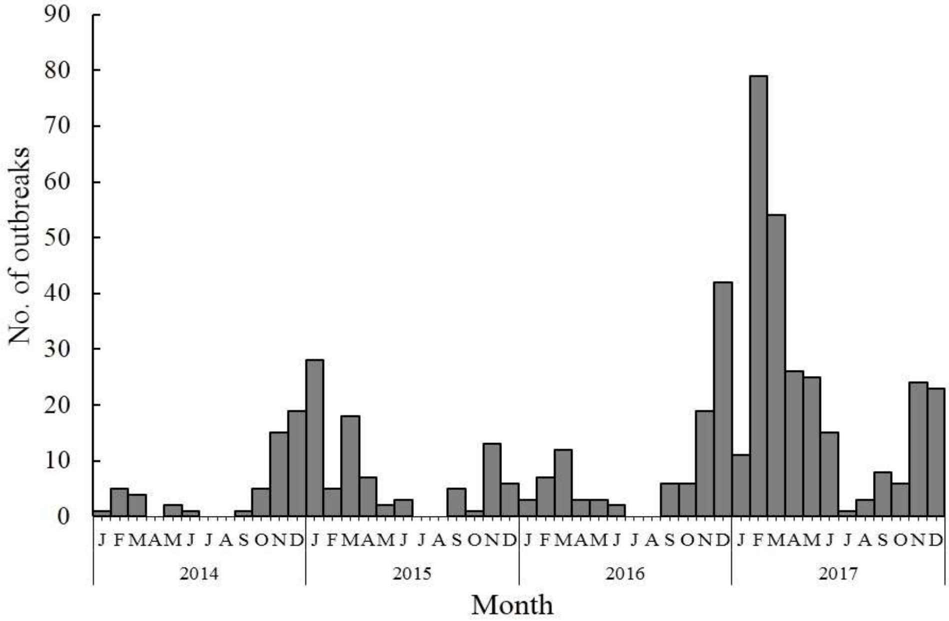

(A)

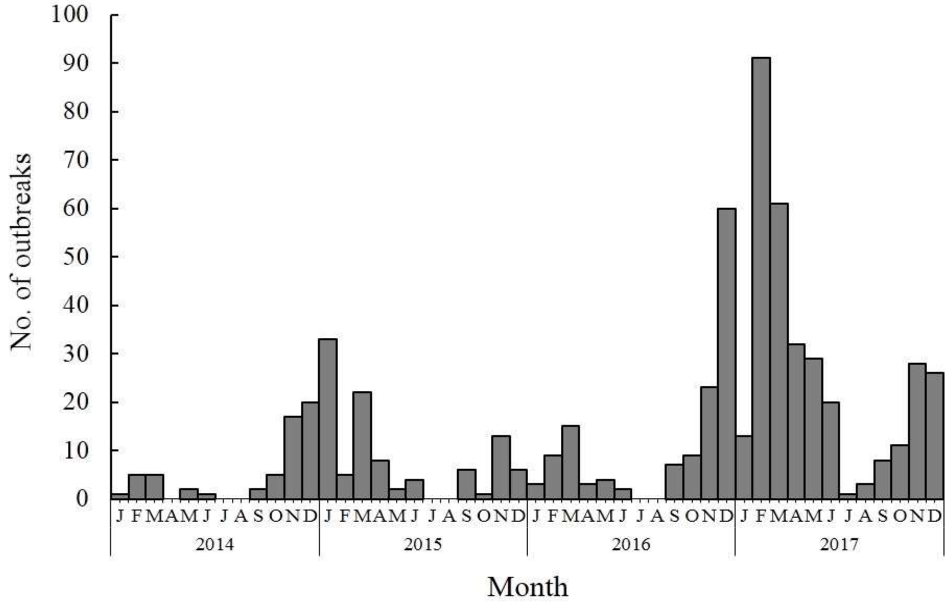

(B)

Figure S1: (**A**) Monthly distribution of norovirus outbreaks involving ≥20 cases reported to the Public Health Emergency Event Surveillance System (PHEESS), China, 2014–2017 (n = 519). (**B**) Monthly distribution of norovirus outbreaks involving <20 cases reported to the Public Health Emergency Event Surveillance System (PHEESS), China, 2014–2017 (n = 97).

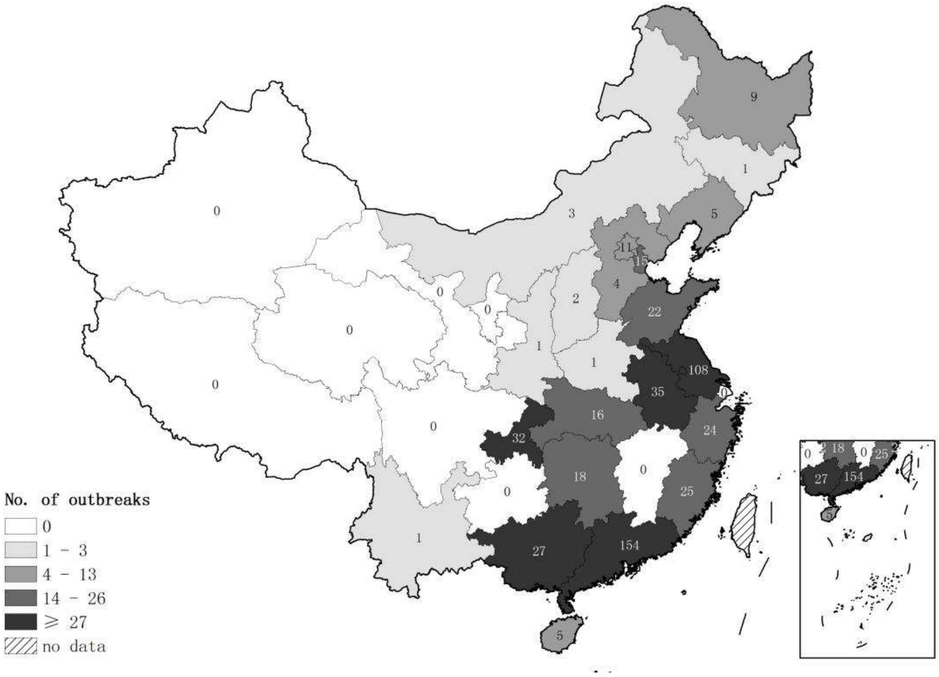

(A)

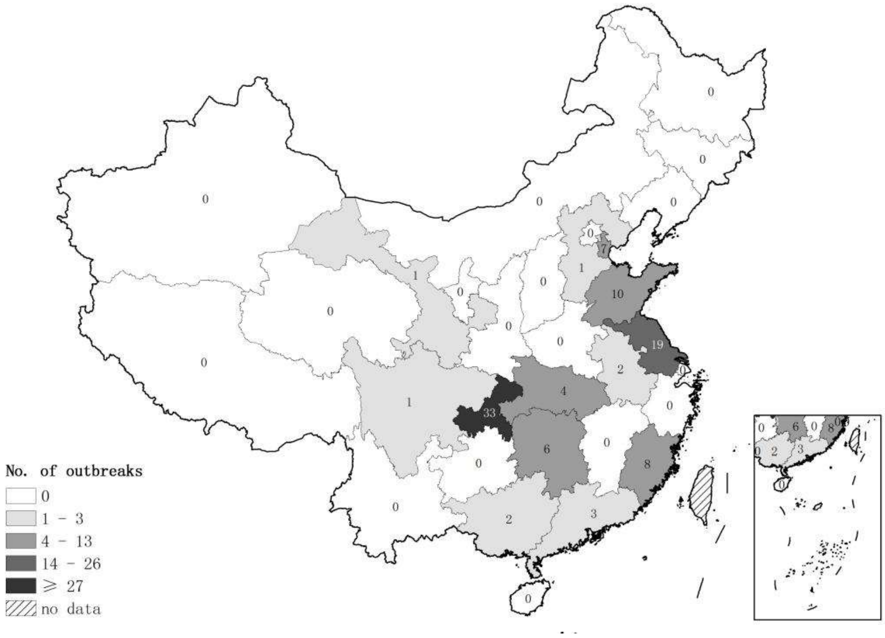

(B)

Figure S2: (**A**) Number of norovirus outbreaks involving ≥20 cases reported to the Public Health Emergency Event Surveillance System (PHEESS) by province, China, 2014–2017 (*n* =519). (**B**) Number of norovirus outbreaks involving <20 cases reported to the Public Health Emergency Event Surveillance System (PHEESS) by province, China, 2014–2017 (*n* = 97).
